# Human Neuromuscular System Identification Using Functional Electrical Stimulation for the Development of a Digital Twin of the Locomotor System

**DOI:** 10.7759/cureus.95270

**Published:** 2025-10-23

**Authors:** Soichiro Hori, Kazuhiro Matsui, Keita Atsuumi, Yoshiki Mori, Hiroaki Hirai, Atsushi Nishikawa

**Affiliations:** 1 Graduate School of Engineering Science, The University of Osaka, Toyonaka, JPN; 2 Faculty of Information Science and Arts, Osaka Electro-Communication University, Shijonawate, JPN; 3 Graduate School of Information Sciences, Hiroshima City University, Hiroshima, JPN

**Keywords:** equilibrium point hypothesis, functional electrical stimulation (fes), human digital twin, neuromusculoskeletal system, system identification

## Abstract

Introduction

Our research group has been developing and applying a human digital twin of the locomotor system and has proposed a simple method for estimating the dynamics of the neuromusculoskeletal system using functional electrical stimulation based on the equilibrium point hypothesis, which focuses on coordination between extensor and flexor muscles. This method defines two parameters: the electrical agonist-antagonist ratio (*r*_E_) and sum (*s*_E_), representing the ratio and sum of the stimulation intensities applied to the extensor and flexor muscles, respectively. Our previous study showed that the relationship between *r*_E_ and the evoked force, i.e., the neuromuscular system (NMS), can be approximated by a second-order system with dead time under isometric conditions, and that the NMS parameters vary with *s*_E_. However, this variation has not yet been modeled. This study investigates how *s*_E_ influences the parameters of isometric elbow joint motion with one degree of freedom.

Methods

Under isometric contraction, we conducted experiments to estimate the parameters of a second-order system, such as proportional gain (*K*_p_), natural frequency (*ω*_n_), and damping ratio (*ζ*), at 15 different *s*_E_ levels. Data were collected from 10 participants (nine males and one female; mean age: 22.7 ± 0.8 years; all right-handed). For group-averaged and individual data, we fitted models describing the relationship between *s*_E_ and each parameter. Model performance was evaluated using the corrected Akaike information criterion (AICc) across linear, quadratic, and exponential models.

Results

For *K*_p_, the quadratic model with a concave shape best fit the group mean data as indicated by the AICc values (linear: -2.78, quadratic: -15.8, and exponential: -14.9). For *ω*_n_ and *ζ*, the convex quadratic models best described the group mean (for *ω*_n_, linear: 3.30, quadratic: -2.74, and exponential: 3.46; for *ζ*, linear: -49.8, quadratic: -53.5, and exponential: -49.8). However, at the individual level, some participants exhibited monotonic trends.

Discussion

For *K*_p_, although the quadratic model provided the best fit for the group mean, the exponential model showed comparable AICc values. Moreover, when summing AICc values across individuals, the exponential model yielded the lowest AICc sum, suggesting that the relationship between *K*_p_ and *s*_E_ can be reasonably approximated by an exponential function. For *ω*_n_ and *ζ*, the overall trend with *s*_E_ was best described by a convex quadratic function. However, due to interindividual differences in muscle properties, some participants did not exhibit a turning point within the tested *s*_E_ range, resulting in monotonic trends. These convex patterns may be explained by the influence of the refractory period of skeletal muscle fibers.

Conclusions

The clinical significance of the model obtained in this study lies in its potential to contribute to the development of the human digital twin of the locomotor system. By incorporating dynamics in which each parameter changes in real time with *s*_E_​, it may become possible to estimate human movement from electromyographic (EMG) signals. However, because the stimulation frequency used in this study was higher than the EMG frequency, the influence of the refractory period may have been amplified. Future studies should investigate whether similar parameter trends are observed at stimulation frequencies closer to those of EMG signals.

## Introduction

In recent years, efforts have been made in the medical and engineering fields to develop a human digital twin of the locomotor system to simulate muscle activity [1]. To achieve this, acquiring the dynamics of the targeted neuromusculoskeletal system (NMSS) is essential. In this context, imaging techniques such as computed tomography and magnetic resonance imaging can be used to obtain the anatomical and structural characteristics of the musculoskeletal system (MSS) [2]. However, the reliance of these techniques on large-scale equipment and complex, time-consuming procedures limits their practicality for acquiring dynamic properties.

When simulations based on such imaging data attempt to model muscle contractions without considering intermuscular coordination, the computational cost can become prohibitively high due to the complexity of modeling each muscle independently.

To address these challenges, our research group proposes a method based on the equilibrium point hypothesis that utilizes functional electrical stimulation (FES) with a focus on the coordination between the flexor and extensor muscles [3,4]. This approach enables simple and minimally invasive acquisition of NMSS dynamics and reduces computational demands.

This work is conducted with a long-term goal of integrating the NMSS dynamics data obtained through this method into muscle activity-driven avatars [5,6], thereby contributing to the realization and the widespread adoption of the human digital twin of the locomotor system.

Neuromuscular system (NMS) identification based on the equilibrium point hypothesis

The equilibrium point hypothesis is a prominent theory in the motor physiology field. It posits that for each pair of antagonistic muscles, muscle activity adjusts natural muscle length, thereby regulating the joint angle at equilibrium (i.e., the equilibrium point) [7]. Models based on this hypothesis suggest that human motor control is achieved through two types of commands: one that controls the equilibrium point and another that regulates joint stiffness.

Supporting this theory, Humphrey conducted electrical stimulation experiments in monkeys and identified two distinct cortical neuron populations: stimulation of one group induced joint movement, and stimulation of the other group elicited changes in joint stiffness [8]. These findings provide neurophysiological evidence for the separate control of joint position and stiffness in the motor system.

Through the electromyographic (EMG) analysis of the limbs, our research group previously showed that the ratio of extensor to flexor activity (agonist-antagonist (AA) muscle ratio) corresponds to the equilibrium point, whereas the sum of their activities (AA muscle sum) is related to joint stiffness [9,10]. Building on this concept, we proposed using electrical stimulation intensity as a substitute for muscle activity and introduced two new parameters: the electrical AA muscle (EAA) ratio (*r*_E_), representing the ratio of stimulation intensities applied to the extensor and flexor muscles, and the EAA sum (*s*_E_), representing the sum of stimulation intensities.

Within this framework, the relationship between *r*_E_ and joint angle changes can be described using an NMSS model. In this model, the NMS that maps *r*_E_ to force *F* and the MSS that maps *F* to joint angle *θ* are connected in a cascade. The NMS is approximated as a second-order system with a dead time under isometric conditions. The MSS is approximated as a second-order system. We previously reported that the dynamic characteristics of these systems vary as a function of* s*_E_ [3,4].

While our group has previously examined how the NMS parameters change with *s*_E_ variations, some parameters exhibited inconsistent trends and deviated from our previous findings [11].

This study estimates each NMS parameter, approximated as a second-order system, more precisely by expanding the frequency range of the *r*_E_ input beyond that used in previous studies. Additionally, this study aims to investigate (i) how *s*_E_ influences the parameters of isometric elbow joint motion with one degree of freedom and (ii) whether consistent trends in the change of each parameter as *s*_E_ changes can be observed across multiple participants.

Note that the dead time L was excluded from the present model. This decision was made considering the practical implementation in EMG-driven avatar applications, where varying L according to muscle activation levels may compromise system stability [12]. In addition, prior studies approximated the NMS using second-order models without incorporating the dead time [13,14]. Therefore, in this study, the NMS was modeled as a second-order system without a dead time component.

A preliminary analysis of the experimental data used herein was presented at the 25th Annual Conference of the Society of Instrument and Control Engineers System Integration Division (SI2024), held from December 18 to 21, 2024 [15].

However, the present study comprises a comprehensive reanalysis of models describing the relationships between *s*_E_ and each parameter. It incorporates substantial methodological revisions, including refinement of outlier removal procedures and the introduction of a model selection criterion based on the corrected Akaike information criterion (AICc), which evaluates the goodness of fit and the parsimony of these models [16].

## Materials and methods

Experimental procedure

**Figure 1:**
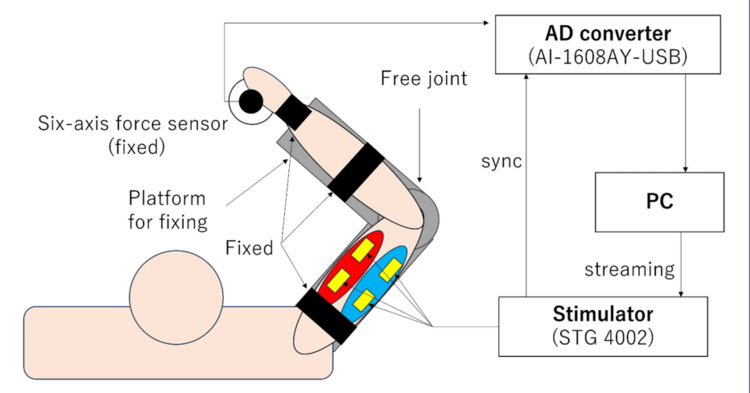
Experimental environment diagram

Twelve healthy adults (11 males, one female; mean age 22.9 ± 0.9 years; all right-handed) participated in the experiment. Figure 1 displays a schematic of the experimental setup.

Isometric conditions were maintained using a custom fixture consisting of a base unit that restricted the joint degrees of freedom (except for the elbow), allowed positional adjustment, and provided gravity compensation, as well as an arm unit for fixing the force sensor. The base height was adjusted individually to ensure each participant adopted a natural and comfortable posture, minimizing fatigue-related effects throughout the experiment.

The wrist joint was immobilized using a wrist supporter reinforced with lightweight metal plates on both sides. The palm was connected to a force sensor mounted on the arm unit, and the upper arm and forearm were fixed to the base. This setup established an isometric contraction environment, allowing elbow joint rotation in the horizontal plane with only one degree of freedom. A six-axis force sensor (PFS080YA501G6, Leptrino Co., Ltd., Komoro, Japan) was used to measure endpoint force. Sensor signals were digitized using an analog-to-digital converter and recorded through a data acquisition system (AI-1608AY-USB, Contec Co., Ltd., Osaka, Japan). The extension direction was defined as the positive direction. For electrical stimulation, a control program was developed in LabVIEW (NI), and the STG4002 stimulator (Multi Channel Systems) was used. For safety, the stimulator was configured to enable rapid power shutdown via a switch in case of emergencies. The target muscles were the right biceps brachii (flexor) and the lateral head of the triceps brachii (extensor). Before both electrode placements, a conductive gel (Compex, TELIC, S.A.U., Barcelona, Spain) was applied to the skin to reduce impedance. Two participants were excluded from the final analysis: one due to reported pain during stimulation and the other due to mild involuntary contractions persisting after stimulation ceased. Consequently, data from 10 participants (nine males and one female; mean age: 22.7 ± 0.8 years; all right-handed) were used.

This study was conducted with the approval of the Ethics Committee for Human Research at the Graduate School of Engineering Science, The University of Osaka (approval R3-3-2).

NMS model identification via FES based on the equilibrium point hypothesis

To exclude low stimulation current levels that failed to elicit detectable muscle responses and to minimize characteristic output differences between the extensor and flexor muscles, we applied normalization to the stimulation intensities.

The minimum force at which muscle contraction was considered to have started was defined as *F*_min_(N), and the maximum endpoint force was defined as *F*_max_(N), respectively. When only the extensor muscle was stimulated, the stimulation current values corresponding to *F*_min_ and *F*_max_ were defined as *I*_emin_(mA) and *I*_emax_(mA), respectively. Similarly, for the flexor muscle, the corresponding stimulation current values were defined as *I*_fmin_(mA) and *I*_fmax_(mA). A 1000 Hz sine wave was used as the carrier signal, and its current amplitude was increased in 0.5 mA increments until the force value reached *F*_max_. In this experiment, *F*_min_ and *F*_max_ were set to 0.5 and 5 N, respectively.

Using the normalized stimulation intensities *I*_e_ and *I*_f_, we defined the *r*_E_ and the *s*_E_ as shown in Equations (1), (2), (3), and (4), respectively.

$I_{\rm e}=\frac{I'_{\rm e}-I_{\rm emin}}{I_{\rm emax}-I_{\rm emin}}$, (1)

$I_{\rm f}=\frac{I'_{\rm f}-I_{\rm fmin}}{I_{\rm fmax}-I_{\rm fmin}}$, (2)

$r_{\rm E}=\frac{I_{\rm e}}{I_{\rm e}+I_{\rm f}}$, (3)

$s_{\rm E}=I_{\rm e}+I_{\rm f}$, (4)

Here, *I'*_e_ and *I'*_f_ are the actual stimulation current values applied to the extensor and flexor muscles, respectively.

**Figure 2:**
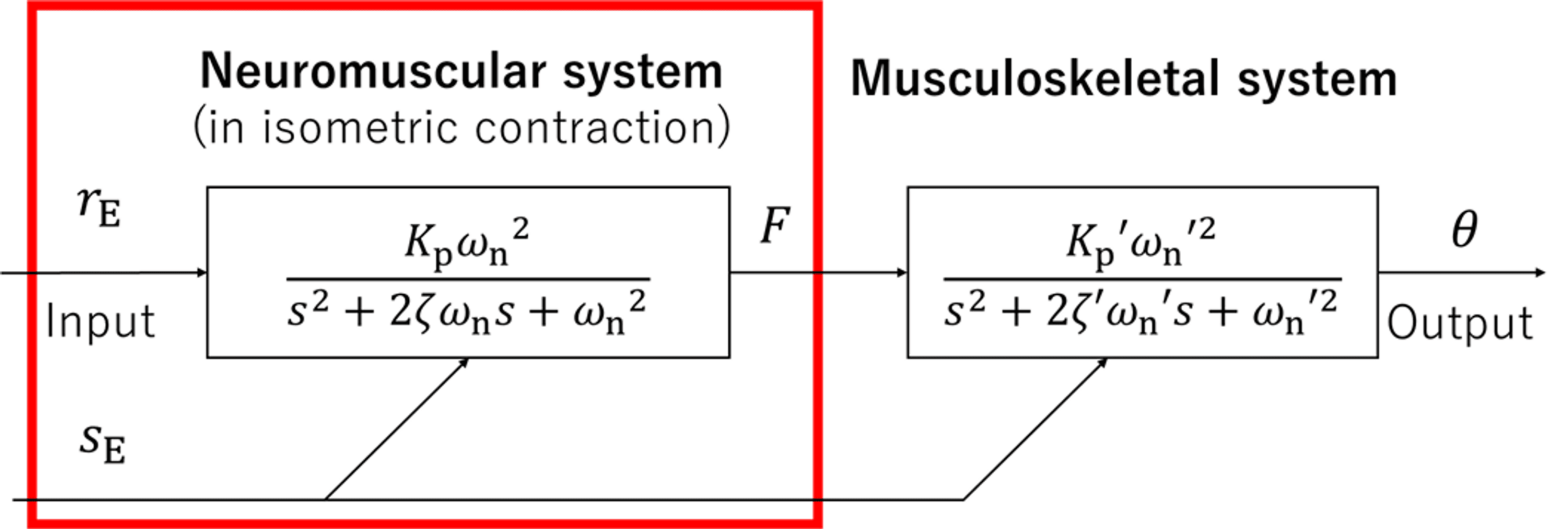
Block diagram of the neuromusculoskeletal system model The red frame indicates the NMS model. NMS, neuromuscular system

Under isometric contraction conditions, we varied *r*_E_ while keeping *s*_E_ constant to derive the input-output relationship of the system while maintaining constant system parameters. The frequency characteristics between the resulting endpoint force *F* and the input *r*_E_ were used to approximate the dynamics of the NMS using a second-order system, as shown in Figure 2. The parameters of this model were then identified.

Electrical stimulation

**Figure 3:**
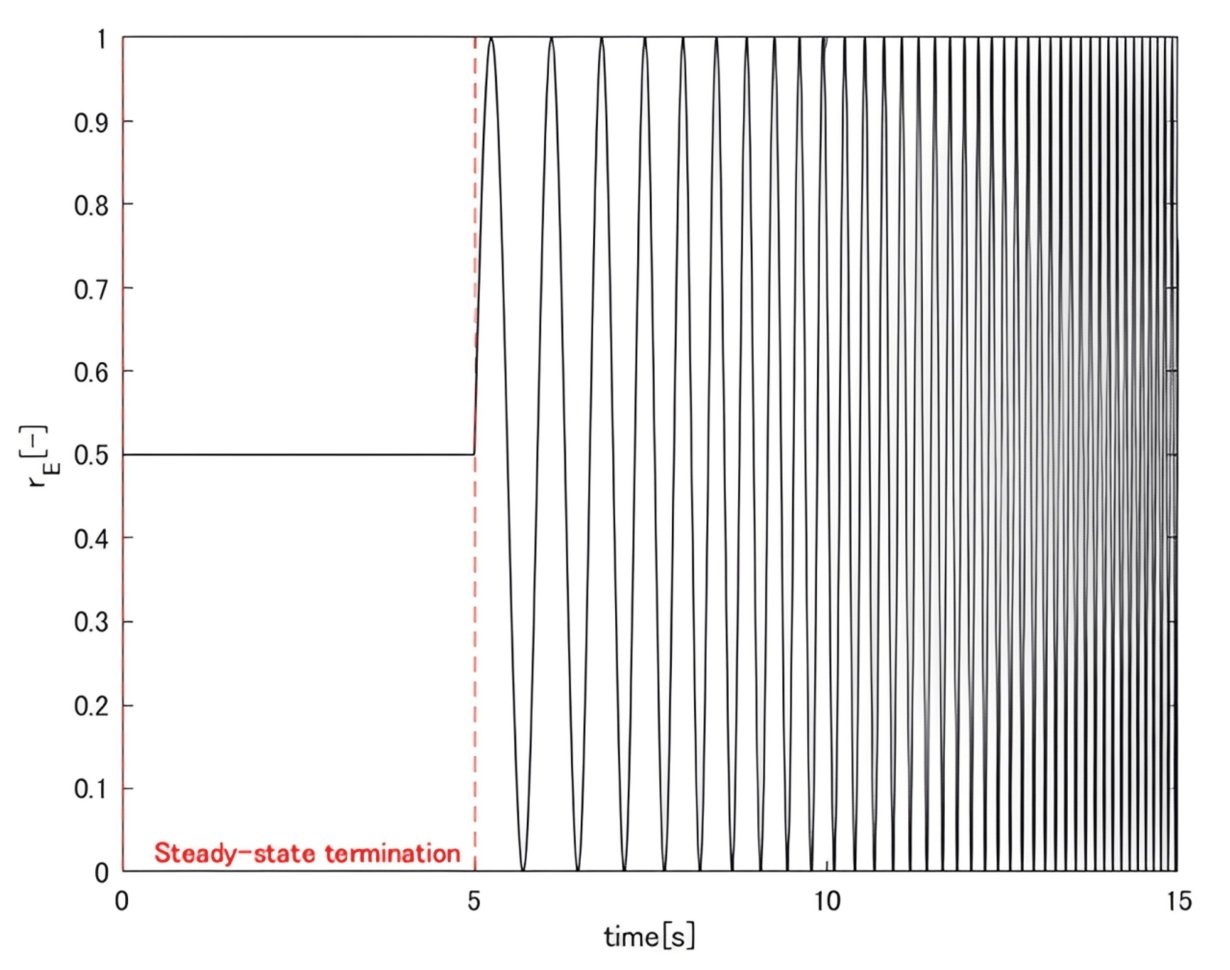
Input rE

A 1000 Hz sine wave was used as the carrier signal and applied under current-controlled conditions. The stimulation intensity was modulated by adjusting the amplitude of this carrier wave. Under the constant *s*_E_ condition, a five-second period with *r*_E_ = 0.5 was introduced to bring the muscles to a steady state, followed by the application of a chirp-like input pattern to *r*_E _(Figure 3).

In this input, *r*_E_ oscillated around 0.5 with an amplitude of 0.5, and its frequency increased exponentially from 1 to 10 Hz over a 10-second interval. The endpoint force was recorded at a sampling frequency of 2000 Hz.

This trial was repeated for 15 different *s*_E_ values ranging from 0.30 to 1.00 in increments of 0.05, with each condition tested three times. To avoid confounding the effect of muscle fatigue with that of changing *s*_E_, the order of *s*_E_ trials was randomized for each participant.

Parameter estimation

Raw force data were first detrended to remove linear drift. The analysis began at the five-second time point, and the corresponding endpoint force signal *F* was extracted. The data were then smoothed using a third-order Savitzky-Golay filter with a window size of 41 samples, followed by noise removal using a low-pass Butterworth filter with a cutoff frequency of 25 Hz.

For each constant *s*_E_, the endpoint forces from the three trials were averaged to compute the mean endpoint force *F*_AVG_. The dynamic relationship between the input *r*_E_ and the mean endpoint force *F*_AVG_ was approximated using a second-order transfer function model. Three parameters were estimated from this model: gain *K*_p_, natural angular frequency *ω*_n_, and damping ratio *ζ*, along with the fitting percentage, which represents the goodness of fit between the estimated model and the actual data. All signal processing and parameter estimation were performed using MATLAB R2023a (MathWorks, Inc., Natick, MA, USA).

Model selection

After parameter estimation, we modeled the relationship between *s*_E_ and each parameter for all participants, as well as the relationship between the participant-averaged parameters and *s*_E_. Three types of models were tested for approximation: linear, quadratic, and exponential. For each, AICc was calculated to comprehensively evaluate the goodness of fit and model parsimony [16]. The model with the lowest AICc value was selected as the best fit. In addition, the sum of AICc values across all participants was computed for each model to determine the most generally applicable model.

However, based on prior research [11], for parameters *ω*_n_ and *ζ*, which tend to be noisier and are less well-modeled than *K*_p_, we first applied outlier removal using the IQR method before performing model selection [17]. After removing individual outliers, participant-wise average values were computed for each parameter, and the IQR method was again applied to the averaged *ω*_n_ and *ζ* values. AICc was then recalculated, and model selection was conducted as described.

## Results

Relationship between *K*_p_ and *s*_E_

**Figure 4:**
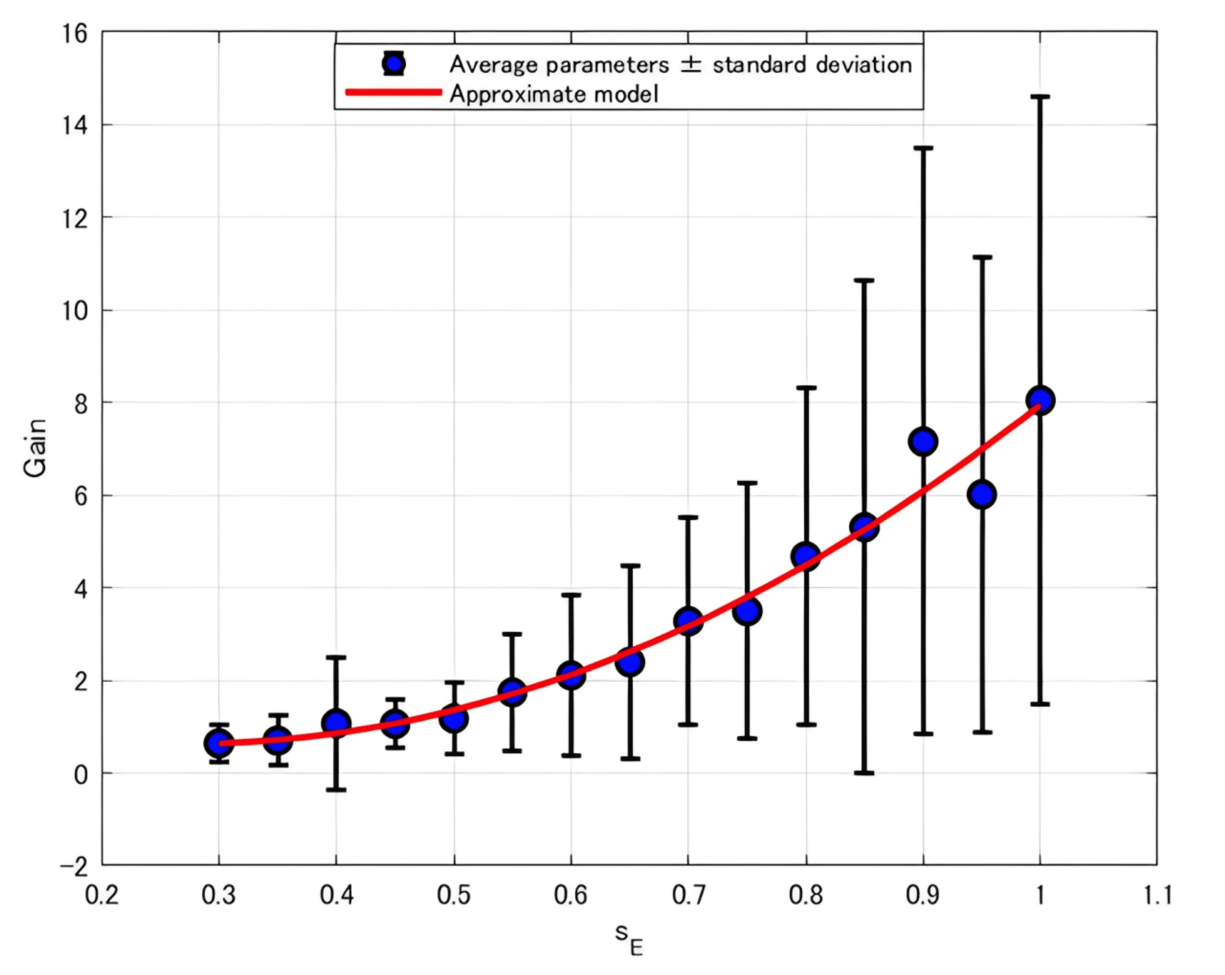
Plot of the average Kp versus sE with the fitted approximation curves

The relationship between *K*_p_ and *s*_E_ for each participant was approximated using three different models: linear, quadratic, and exponential. Table 1 summarizes the corresponding AICc values. The best-fit model for each participant was selected based on the AICc criterion. Table 2 presents the selected best-fit models describing the relationship between *K*_p_ and *s*_E_ for each participant, as well as the model fitted to the averaged *K*_p_ values across participants, along with the general trends of these models, whether they increase or decrease, and whether they are convex or concave. The fitted curve representing the relationship between the average *K*_p_ and *s*_E_ is shown in Figure 4.

**Table 1:** Comparison of AICc values for the three model fits of the relationship between Kp and sE AICc, corrected Akaike information criterion

Participant	Linear model	Quadratic function model	Exponential model
Average	-2.778	-15.76	-14.88
A	38.26	28.63	29.6
B	19.49	23.14	20.82
C	7.03	10.81	9.154
D	-2.804	0.9892	-2.593
E	15.17	16.31	19.15
F	9.174	12.43	8.924
G	-16.73	-20.6	-22.87
H	39.23	42.15	38.92
I	-0.1731	-11.2	-12.22
J	34.59	34.13	28.15

**Table 2:** Selected best-fit models for the relationship between Kp and sE

Participant	Selected approximate model	Trend
Average	Quadratic function model	Concave
A	Quadratic function model	Concave
B	Linear model	Increase
C	Linear model	Increase
D	Linear model	Increase
E	Linear model	Increase
F	Exponential model	Increase
G	Exponential model	Increase
H	Exponential model	Increase
I	Exponential model	Increase
J	Exponential model	Increase

Table 3 shows the sum of the AICc values across all participants for each approximation model of the relationship between *K*_p_ and *s*_E_.

**Table 3:** Sum of the AICc values for each approximation model of the relationship between Kp and sE AICc, corrected Akaike information criterion

Approximate model	Sum of AICc
Linear model	143.2
Quadratic function model	136.8
Exponential model	117.0

All participants exhibited a general tendency for *K*_p_ to increase with increasing *s*_E_. Although the concave quadratic model was selected as the best-fit model for the participant-averaged data, it was not necessarily the best fit for individual participants. When considering the sum of AICc values across all participants, the exponential model exhibited the lowest overall AICc, followed by the quadratic model.

Relationship between *ω*_n_ and *s*_E_

**Figure 5:**
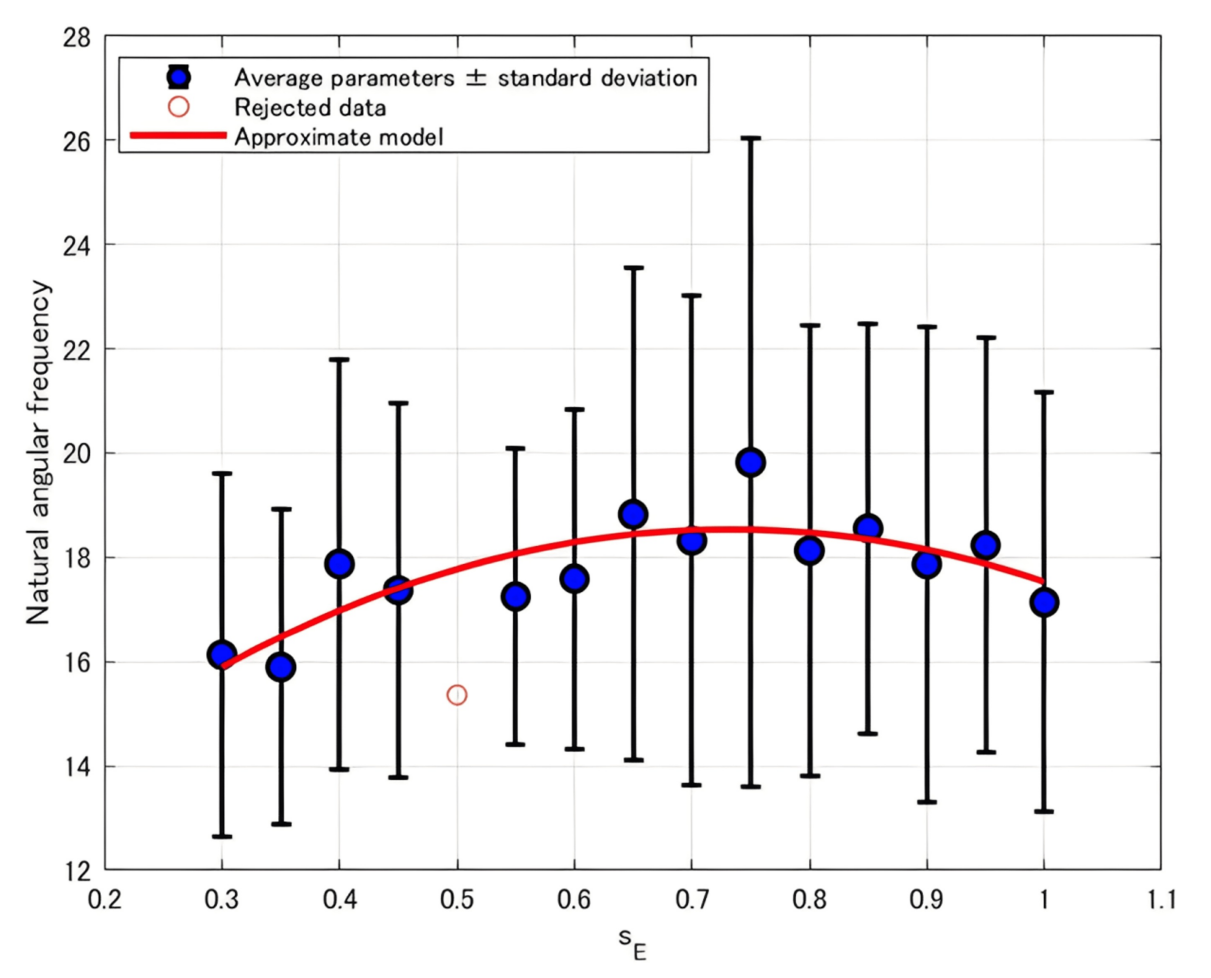
Plot of the average ωn versus sE with the fitted approximation curves

The relationship between *ω*_n_ and *s*_E_ for each participant was approximated using three different models. Table 4 summarizes the corresponding AICc values. The best-fit model for each participant was identified based on the AICc criterion. Table 5 presents the selected best-fit models describing the relationship between *ω*_n_ and *s*_E_ for each participant, as well as the model fitted to the averaged *ω*_n_ values across participants, along with the general trends of these models. Figure 5 depicts the fitted curve representing the relationship between the average *ω*_n_ and *s*_E_.

**Table 4:** Comparison of AICc values for three model fits of the relationship between ωn and sE ​AICc, corrected Akaike information criterion

Participant	Linear model	Quadratic function model	Exponential model
Average	3.297	-2.744	3.460
A	24.13	26.35	24.15
B	12.63	16.63	12.59
C	22.918	20.63	22.917
D	31.27	33.58	31.23
E	22.5295	24.63	22.5298
F	37.14	36.75	37.64
G	38.56	36.52	39.54
H	30.84	30.79	30.17
I	45.33	43.96	45.53
J	45.67	45.65	45.71

**Table 5:** Selected best-fit models for the relationship between ωn and sE

Participant	Selected approximate model	Trend
Average	Quadratic function model	Convex
A	Linear model	Decrease
B	Exponential model	Increase
C	Quadratic function model	Concave
D	Exponential model	Increase
E	Linear model	Decrease
F	Quadratic function model	Convex
G	Quadratic function model	Convex
H	Exponential model	Decrease
I	Quadratic function model	Convex
J	Quadratic function model	Convex

Table 6 presents the sum of the AICc values across all participants for each approximation model of the relationship between *ω*_n_ and *s*_E_.

**Table 6:** Sum of the AICc values for each approximation model of the relationship between ωn and sE AICc, corrected Akaike information criterion

Approximate model	Sum of AICc
Linear model	311.0
Quadratic function model	315.5
Exponential model	312.0

For *ω*_n_, when averaging the parameter values across all participants, the best-fit model was a convex quadratic function (Figure 5). However, as shown in Table 5, the selected models for individual participants varied. Some participants exhibited a monotonic increase or decrease, either linear or nonlinear, with increasing *s*_E_, while the data from others were better approximated by a model that included a peak point. This type of behavior, where the parameter reaches a peak in response to increasing *s*_E_, has not been reported in previous studies [11].

In contrast, Table 6 shows that when considering the sum of AICc values across all participants, the linear model yielded the lowest AICc sum, whereas the quadratic model resulted in the highest.

Relationship between *ζ* and *s*_E_

**Figure 6:**
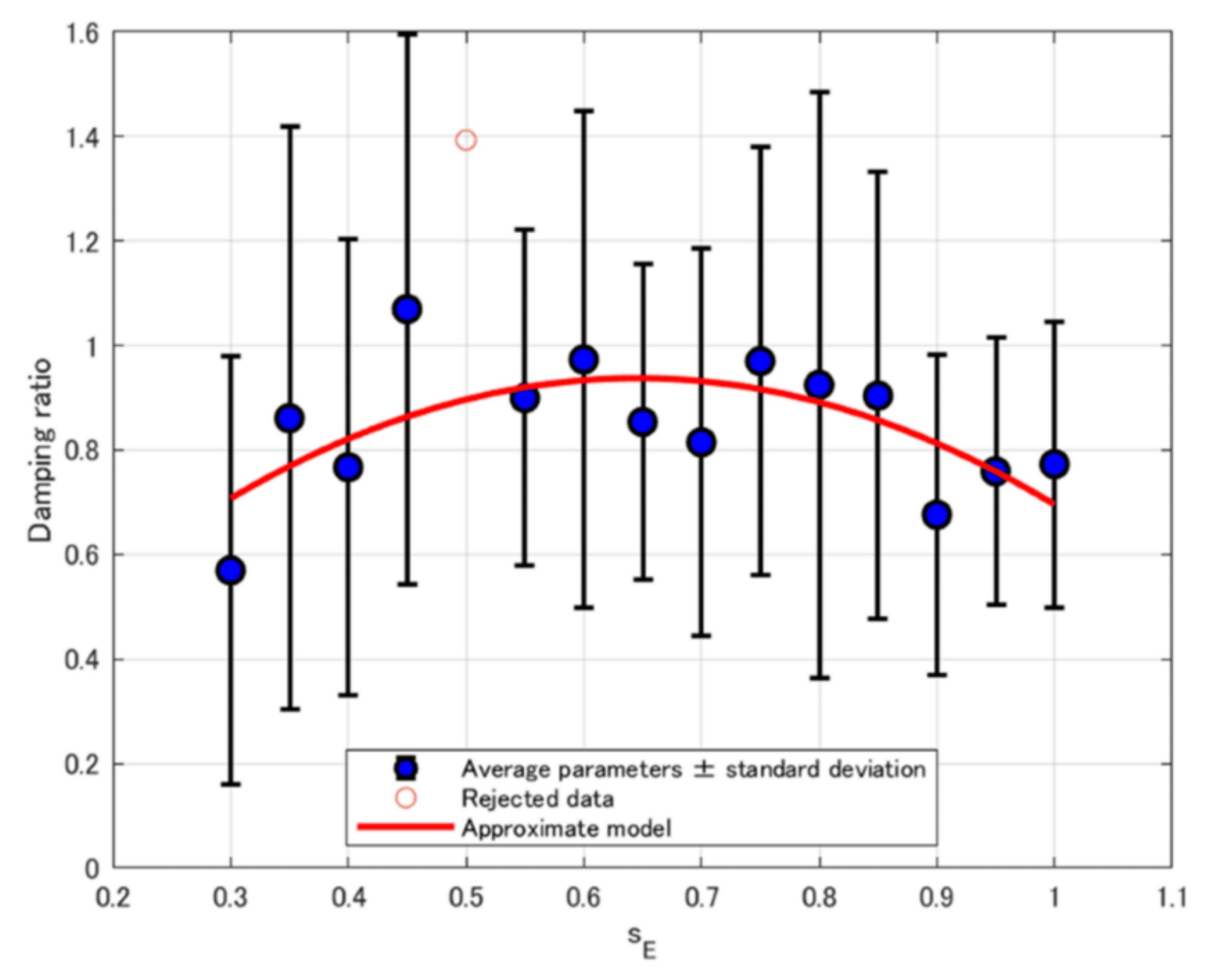
Plot of the average ζ versus sE with fitted approximation curves

The relationship between *ζ* and *s*_E_ for each participant was approximated using three different models. Table 7 summarizes the corresponding AICc values. The best-fit model for each participant was identified based on the AICc criterion. Table 8 presents the selected best-fit models describing the relationship between *ζ* and *s*_E_ for each participant, as well as the model fitted to the averaged *ζ* values across participants, along with the general trends of these models. Figure 6 displays the fitted curve representing the relationship between the average *ζ* and *s*_E_.

**Table 7:** Comparison of AICc values for three model fits of the relationship between ζ and sE AICc, corrected Akaike information criterion

Participant	Linear model	Quadratic function model	Exponential model
Average	-49.77	-53.48	-49.77
A	-29.15	-33.50	-28.15
B	-34.13	-34.05	-34.93
C	-52.64	-51.77	-52.59
D	-30.73	-26.92	-30.75
E	-24.37	-21.24	-24.32
F	-21.97	-20.87	-23.81
G	-27.05	-25.08	-26.84
H	-41.73	-42.26	-41.42
I	16.82	21.04	16.48
J	-37.85	-34.70	-37.76

**Table 8:** Selected best-fit models for the relationship between ζ and sE

Participant	Selected approximate model	Trend
Average	Quadratic function model	Convex
A	Quadratic function model	Convex
B	Exponential model	Decrease
C	Linear model	Increase
D	Exponential model	Decrease
E	Linear model	Increase
F	Exponential model	Decrease
G	Linear model	Decrease
H	Quadratic function model	Convex
I	Exponential model	Decrease
J	Linear model	Increase

Table 9 shows the sum of the AICc values across all participants for each approximation model of the relationship between *ζ* and *s*_E_.

**Table 9:** Sum of the AICc values for each approximation model of the relationship between ζ and sE AICc, corrected Akaike information criterion

Approximate model	Sum of AICc
Linear model	-282.8
Quadratic function model	-269.4
Exponential model	-284.1

For *ζ*, similar to the *ω*_n_ results, when the parameter values were averaged across participants, the best-fit model was a convex quadratic function (Figure 6). However, as shown in Table 8, the selected models for individual participants varied. Some participants exhibited a monotonic increase or decrease, either linear or nonlinear, with increasing *s*_E_, while the data from others were better approximated by a model that included a peak point. This type of behavior, where the parameter reaches a peak in response to increasing *s*_E_, has not been reported in previous studies [11].

In contrast, Table 9 shows that when considering the sum of AICc values across all participants, the exponential model yielded the lowest AICc sum, whereas the quadratic model resulted in the highest.

## Discussion

Trend in *K*_p_

As noted in the Results section, all participants exhibited a tendency for the selected model to show an increasing trend in *K*_p_ with the increasing *s*_E_, confirming that *K*_p_ monotonically increases as *s*_E_ increases. Because *K*_p_ represents the output magnitude in response to input (i.e., the magnitude of muscle force evoked by electrical stimulation), this trend aligns with the intuitive expectation that stronger stimulation to flexor and extensor muscles (via increased *s*_E_) would induce greater force.

As shown in Table 3, when evaluating the sum of AICc values across all participants, the exponential model yielded the lowest value, followed by the quadratic model, suggesting that nonlinear models provided better fits than the linear model.

Furthermore, we examined the number of cases in which the AICc was the highest, indicating that the model was evaluated as the least appropriate among the three approximation models. The linear model was evaluated as the least appropriate model for four out of 10 participants, the quadratic model for five out of 10 participants, and the exponential model for only one out of 10 participants.

These findings suggest that *K*_p_ exhibits a nonlinear increasing trend with respect to *s*_E_, and that the exponential model provides the most appropriate approximation of this relationship. This result is consistent with findings reported in previous studies [11].

Trend in *ω*_n_ and *ζ*

As described in the Results section, *ω*_n_ and *ζ* were best approximated by a convex quadratic model when averaged across participants, as shown in Figure 5 and Figure 6. However, as detailed in Table 5 and Table 8, the individual participant trends varied: while some showed a monotonic trend increase or decrease with increasing *s*_E_, others exhibited relationships with a turning point, indicating the presence of a local extremum. This variation likely contributed to the larger AICc sum values of quadratic models than those of linear models and exponential models, as shown in Table 6 and Table 9.

These results suggest that the relationships between *s*_E_ and *ω*_n_ or *ζ* fundamentally follow a convex quadratic model, with each parameter reaching an extremum at a certain *s*_E_ value. For participants whose data showed a monotonic trend, it is likely that the extremum lay outside the tested stimulation range due to individual differences in muscle properties.

A potential explanation for the presence of a turning point is the influence of the refractory period of the skeletal muscle fibers [18]. As *s*_E_ increases, more muscle fibers become responsive to electrical stimulation. However, the number of fibers in the refractory state also increases. This interplay between increased recruitment and reduced responsiveness due to refractoriness may underlie the observed nonlinear trend.

The influence of the refractory period is closely related to stimulation frequency. In this study, we used a carrier frequency of 1000 Hz was used to reduce perceived discomfort. However, this frequency is substantially higher than the typical frequency range of human EMG signals (approximately 10-500 Hz) [19]. As such, the stimulation frequency exceeded the physiological firing rate of muscle fibers during voluntary movement. This high-frequency stimulation may have amplified the refractory period effects, making the decline in responsiveness more prominent than voluntary muscle activity in humans. Therefore, further investigation is needed to determine whether similar parameter trends would be observed using stimulation frequencies closer to the physiological range.

## Conclusions

Herein, we investigated how the dynamic parameters of the elbow joint change in response to variations in *s*_E_, using an NMS identification method based on FES grounded in the equilibrium point hypothesis. This method provides a simple yet effective approach to estimating joint dynamics and contributes to the development of the human digital twin of the locomotor system as a clinically significant application. By incorporating a dynamic model in which parameters change in real time according to *s*_E_, it may become possible to compute human motion from EMG signals within a digital twin framework. *K*_p_ exhibited a nonlinear increasing trend with respect to *s*_E_, and the exponential model was found to best represent the relationship between *K*_p_ and *s*_E_. This finding is consistent with those of previous studies. In contrast, for *ω*_n_ and *ζ*, the averages across participants were best approximated by a convex quadratic model. However, when analyzing individual data, no single consistent trend emerged. Some participants showed linear relationships, while others exhibited nonlinear trends with turning points. These results suggest that the relationships between *s*_E_ and *ω*_n_, *ζ* are inherently convex quadratic models involving a local extremum. For participants with parameters that appeared to vary monotonically, the extremum likely lies outside the range of stimulation intensities applied in this study.

We hypothesize that these nonlinear relationships arise from the interplay between two opposing effects: the increasing number of responsive muscle fibers as the stimulation intensity increases and the growing influence of the muscle fiber refractory period, which decreases the responsiveness. This shift in dominance between facilitation and suppression may explain the observed turning points. However, the effects of the refractory period are strongly influenced by the stimulation frequency, and this study employed frequencies higher than physiological rates; hence, future studies should examine whether similar parameter trends are observed at stimulation frequencies closer to those of natural EMG signals.

## References

[1] Saxby DJ, Pizzolato C, Diamond LE (2023). A digital twin framework for precision neuromusculoskeletal health care: extension upon industrial standards. J Appl Biomech.

[2] Yokota F, Otake Y, Takao M, Ogawa T, Okada T, Sugano N, Sato Y (2018). Automated muscle segmentation from CT images of the hip and thigh using a hierarchical multi-atlas method. Int J Comput Assist Radiol Surg.

[3] Matsui K, Hishii Y, Maegaki K, Yamashita Y, Uemura M, Hirai H, Miyazaki F (2014). Equilibrium-point control of human elbow-joint movement under isometric environment by using multichannel functional electrical stimulation. Front Neurosci.

[4] Suzuki Y, Matsui K, Atsuumi K, Taniguchi K, Hirai H, Nishikawa A (2024). Feasibility of human wrist-joint neuromuscular system identification method using functional electrical stimulation in clinical examinations. Adv Biomed Eng.

[5] Okamoto Y, Matsui K, Ando T, Atsuumi K, Taniguchi K, Hirai H, Nishikawa A (2024). Pilot study of the relation between various dynamics of avatar experience and perceptual characteristics. PeerJ Comput Sci.

[6] Ando T, Matsui K, Okamoto Y, Atsuumi K, Taniguchi K, Hirai H, Nishikawa A (2024). Physio-avatar EB: aftereffects in error learning with EMG manipulation of first-person avatar experience. Front Bioeng Biotechnol.

[7] Feldman AG (1986). Once more on the equilibrium-point hypothesis (λ model) for motor control. J Mot Behav.

[8] Humphrey DR (1982). Separate cell systems in the motor cortex of the monkey for the control of joint movement and of joint stiffness. Electroencephalogr Clin Neurophysiol Suppl.

[9] Iimura T, Inoue K, Pham HT, Hirai H, Miyazaki F (2011). Decomposition of limb movement based on muscular coordination during human running. J Adv Comput Intell Intell Inform.

[10] Ariga Y, Pham HTT, Uemura M, Hirai H, Miyazaki F (2012). Novel equilibrium-point control of agonist-antagonist system with pneumatic artificial muscles. 2012 IEEE International Conference on Robotics and Automation.

[11] Hori S, Matsui K, Atsuumi K, Taniguchi K, Hirai H, Nishikawa A (2025). Tendency of characteristic change by electrical agonist-antagonist muscle sum when using a human neuromuscular system identification method [Article in Japanese]. IEEJ Trans Electron Inf Syst.

[12] Richard JP (2003). Time-delay systems: an overview of some recent advances and open problems. Automatica.

[13] Masani K, Vette AH, Kawashima N, Popovic MR (2008). Neuromusculoskeletal torque-generation process has a large destabilizing effect on the control mechanism of quiet standing. J Neurophysiol.

[14] Uchiyama T, Kondo G (2020). Relationships among electromyogram, displacement and velocity of the center of pressure, and muscle stiffness of the medial gastrocnemius muscle during quiet standing. Adv Biomed Eng.

[15] Hori S, Matsui K, Hirai H, Nishikawa A (2024). Characteristic of dynamics changes induced by electrical agonist-antagonist muscle sum in human neuromuscular system identification. examination of electrical agonist-antagonist muscle ratio input with broad frequency range. Proceedings of the 25th Annual Conference of the Society of Instrument and Control Engineers (SICE), System Integration Division.

[16] (2022). Model Selection and Multimodel Inference: A Practical Information-Theoretic Approach.

[17] Tukey JW (1977). Exploratory Data Analysis, Vol. 2. Exploratory Data Analysis, Vol. 2.

[18] Lateva ZC, McGill KC, Johanson ME (2002). Electrophysiological evidence of adult human skeletal muscle fibres with multiple endplates and polyneuronal innervation. J Physiol.

[19] Muceli S, Merletti R (2024). Tutorial. Frequency analysis of the surface EMG signal: best practices. J Electromyogr Kinesiol.

